# The effect of beta blocker withdrawal on adenosine myocardial perfusion imaging

**DOI:** 10.1007/s12350-014-9952-y

**Published:** 2014-08-15

**Authors:** C. Hoffmeister, R. Preuss, R. Weise, W. Burchert, O. Lindner

**Affiliations:** 1 Diabetes Center, Heart and Diabetes Center North Rhine-Westphalia, University Hospital of the Ruhr-University Bochum, Bad Oeynhausen, Germany; 2 Institute of Radiology, Nuclear Medicine and Molecular Imaging, Heart and Diabetes Center North Rhine-Westphalia, University Hospital of the Ruhr-University Bochum, Bad Oeynhausen, Germany

**Keywords:** Myocardial perfusion imaging, beta blocker, adenosine, PET

## Abstract

**Background:**

The effect of beta blockers on myocardial blood flow (MBF) under vasodilators has been studied in several SPECT and PET myocardial perfusion imaging (MPI) studies with divergent results. The present study evaluated the effect of a beta blocker withdrawal on quantitative adenosine MBF and on MPI results.

**Methods:**

Twenty patients with beta blockers and CAD history were studied with quantitative adenosine N-13 ammonia PET. The first study was performed under complete medication and the second after beta blocker withdrawal. The PET studies were independently read with respect to MPI result and clinical decision making.

**Results:**

Global MBF showed an increase from 180.2 ± 59.9 to 193.6 ± 60.8 mL·minute^−1^/100 g (*P* = .02) after beta blocker withdrawal. The segmental perfusion values were closely correlated (*R* ^2^ = 0.82) over the entire range of perfusion values. An essentially different interpretation after beta blocker discontinuation was found in two cases (10%).

**Conclusion:**

A beta blocker withdrawal induces an increase in adenosine MBF. In the majority of cases, MPI interpretation and decision making are independent of beta blocker intake. If a temporary beta blocker withdrawal before MPI is not possible or was not realized by the patient, it is appropriate to perform adenosine stress testing without loss of the essential MPI result.

## Introduction

Myocardial perfusion imaging (MPI) is an effective noninvasive modality for diagnosing coronary artery disease (CAD) and yields valuable information concerning risk, prognosis and therapeutical management.^1^,^2^ Ergometry is the stress test of choice and most frequently applied. However, the proportion of vasodilator stress tests with adenosine, dipyridamole and recently with regadenoson is growing.^3^ All vasodilators increase MBF to comparable levels.^4^,^5^ Adenosine and regadenoson through direct coronary vasodilation via adenosine A2A receptors, and dipyridamole indirectly through a prolonged action of endogenous adenosine by an inhibition of adenosine deaminase.^6^,^7^

Despite maximal vasodilation by pharmacologic vasodilators, an interaction of cardiac medications such as beta blockers, calcium blockers, and nitrates has been suggested in several studies.^8^-^12^ Current guidelines recommend temporarily discontinuing beta blockers before stress testing.^13^,^14^

In clinical routine a temporary beta blocker withdrawal before stress perfusion testing is sometimes not possible due to complications, contraindications, a tight clinical schedule, or because a patient simply forgot to withhold the medication. Thus, the question arises of whether MPI under vasodilators is affected by beta blockers or their withdrawal at all.

The results of some recent studies addressing this issue are contradictory:

Two retrospective analyses reported a similar sensitivity and specificity between patients with and without beta blockers and no effect of beta blocker therapy on extent, severity, and perfusion defects.^15^,^16^ On the other hand, a significant impact of beta blockers on adenosine MPI has been demonstrated in a prospective study.^12^ All the cited studies related to MPI in SPECT technique.

The present prospective study was designed to determine the absolute effect of a temporary withdrawal of beta blockers on quantitative adenosine MBF with N-13 ammonia PET and to estimate its impact on the results and the interpretation of PET MPI.

## Methods

### Patient Population and Study Protocol

We studied a total of 20 patients (68 ± 11 years, 15 men) with CAD and typical dosage regimens of cardiac drugs, including long-term beta blocker medication (Table 1). Exclusion criteria were contraindications for adenosine stress testing and for discontinuation of beta blocker therapy.

**Table 1:** Patient characteristics

		%
Age	68 ± 11	
Gender	15m, 5f	75/25
Cardiac risk factors		
Diabetes mellitus	15	75
Hyperlipidemia	17	85
Hypertension	18	90
Smoking and ex-smoking	7 (3/4)	(15/20)
Beta blocker		
Bisoprolol	10	50
Metoprolol	5	25
Nebivolol	4	20
Carvedilol	1	5
Other cardiac medication		
Aspirin	19	95
Clopidogrel/prasugrel	8	40
ACE inhibitors	12	60
AT1-receptor antagonists	4	20
Central receptor blockers	3	15
Diuretics	13	65
Calcium antagonists	8	40
Nitrates	6	30
Statins	16	80
Cardiac history		
CAD	18	90
Prior infarction	3	15
Prior PTCA	14	70
Prior CABG	5	25
Cardiomyopathy	2	10
Arrhythmia	1	5

The first imaging procedure was performed under complete medication and consisted of a quantitative adenosine N-13 ammonia PET scan and, if necessary, of an N-13 ammonia rest scan. A second quantitative adenosine N-13 ammonia PET scan, which represented the actual study acquisition, was performed after withdrawal of the individual beta blocker for at least four half-lives. The other cardiac medication remained unchanged.

The study protocol was approved by the local ethics committee of the Ruhr-University Bochum (Reg. No. 42/2011) and the German Federal Office for Radiation Protection (Bundesamt für Strahlenschutz, Z5-22463/2-2011-014). All patients gave their written informed consent.

### Adenosine Stress Testing and Image Acquisition

All patients were investigated with a Biograph mCT (Siemens, Erlangen, Germany). Directly before the PET acquisition a low dose CT scan for attenuation correction was performed. In this paper, the term PET is used instead of PET-CT since we only refer to the PET measurements.

Adenosine was infused intravenously at a constant rate of 0.14 mg·kg^−1^·minute^−1^ over 6 minutes.

Two minutes after the onset of the adenosine infusion, about 600 MBq N-13 ammonia was injected as an intravenous bolus. Image acquisition over 15 minutes was started simultaneously with the bolus injection. Data were recorded in list mode. A consecutive set of 20 frames (12 frames 5 seconds, 5 frames 30 seconds, 2 frames 120 seconds, 1 frame 450 seconds) was reconstructed for quantification of perfusion. The last 5 minutes of the emission scan were used for ECG-gated reconstruction with 12 gates.

### Adenosine Side Effects

The commonest adenosine side effects (flush, headache, thoracic pressure, angina pectoris, and dyspnea) were assessed with a four-category score (0—no symptom, 1—mild, 2—moderate, 3—severe symptoms) in each case and finally summed to a global side-effect score with a range from 0 to 15. A 12-channel ECG was started 1 minute before the adenosine infusion up to the end of the emission scan. Arrhythmias and ST-segment depressions were recorded.

### Quantitative Analysis and Scoring of PET Perfusion Data

Quantification of the N-13 ammonia scans was based on an irreversible 2-compartment model which was implemented in MATLAB.^17^ The model was fitted to the dynamic data using the linear least squares approach first proposed by Blomqvist.^18^ Corrections for fractional blood volume, limited recovery due to partial volume effects and spillover activity from left ventricular blood pool to tissue were performed as described elsewhere.^19^ The implementation of the fitting procedure was validated by using a representative measured arterial input function and the analytical solution of the described 2-compartment model, including all correction terms for generation of simulated tissue response functions at different assumed flow levels. A validation in humans was performed by the argon inert gas technique.^20^ The quantification procedure delivered 20-segment parametric polarmaps of MBF. Segments with a fractional blood volume >0.5 were excluded from further analysis. This value was taken as an empirical cut-off indicating failure to correctly delineate the center of the myocardial wall. Such large values are only explainable by massive spillover from the ventricle and occurred in most cases in the basal segments of the septum. Furthermore, segments with a resting MBF < 50 mL·minute^−1^/100 g were regarded as infarcted and also excluded from the analysis. A total of ten segments in three patients were rejected from analysis for this particular reason. Global perfusion was calculated as the average of all myocardial segments.

### Hemodynamic Parameters

During the stress phase, heart rate and blood pressure were recorded every 2 minutes, starting with the onset of the adenosine infusion until completion of the infusion after 6 minutes. Mean arterial blood pressure was calculated from the average values of all 4-time points and mCR as mean arterial blood pressure/global perfusion. To account for changes in MBF by different cardiac work at the time of the two individual scans, global MBF was normalized to a rate-pressure product (RPP) of 10,000 mm Hg·minute^−1^. The normalization was achieved by multiplying the global MBF value by the ratio of an RPP of 10,000 mm Hg·minute^−1^ and the average RPP during the individual adenosine stress test.

### PET Study Interpretation

The PET study sets were read by two nuclear medicine physicians with expertise in nuclear cardiology and unaware of the status of beta blocker intake. For the interpretation and the management recommendation, the readers considered the quantitative polarmaps with tables of absolute segmental MBF under adenosine, segmental fractional blood volume for quality control, clinical information and, if performed, the rest MBF study.

The graduation of MBF abnormalities included both extent and severity of disturbances. Based on routine clinical judgment, they were classified as normal, mildly, moderately, and severely abnormal. A MBF > 200 mL·minute^−1^/100 g was regarded as normal. The clinical interpretation with patient management recommendation were “risk modification” in normal, “medical therapy” in mild, and “further diagnosis (CT or invasive angiography)” in moderate and severe results.^21^

In special cases where an angiography had been performed shortly before the studies or prescan data were available, the management recommendation was adjusted accordingly.

### Statistical Analysis

For the sample size estimation, a power of 90% and a significance criterion of 0.05 were chosen. The minimum expected differences between the two means and the standard deviation were estimated to 10 mL·minute^−1^/100 g each. Accordingly, about 20 patients had to be enrolled in the study.^22^

Data are given as mean value ± standard deviation. In the first step, the paired parameters were tested for the normal distribution with the Kolmogorov-Smirnov test. As all parameters were normally distributed, post hoc comparisons were performed with a paired *t* test. Differences were considered statistically significant at values <0.05 (two-sided). Pearson’s correlation coefficient was used to assess the interrelationship between absolute MBF with and without beta blocker use. For the analyses, the statistical software package IBM SPSS (version 20) was used.

## Results

### Clinical Characteristics, Hemodynamic Parameters, and Ventricular Function

Table 1 summarizes the characteristics of the study patients. Nineteen patients (95%) took cardioselective beta blockers and one patient (5%) the non cardioselective beta blocker carvedilol.

The beta blocker withdrawal was well tolerated by all patients without exacerbation of angina symptoms.

Hemodynamic parameters are given in Table 2 (upper third). Mean systolic and mean diastolic blood pressure during adenosine were nearly identical (*P* = .77 and *P* = .78) with and without beta blocker. Mean heart rate and mean RPP during adenosine significantly increased after beta blocker withdrawal by 17% ± 17% (*P* < .001) and 19% ± 23% (*P* = .004), respectively.

**Table 2:** Hemodynamic response under adenosine, perfusion, and left-ventricular function

	With beta blocker	Without beta blocker	*P*
Heart rate (BPM)	69.7 ± 12.1	80.3 ± 10.9	<.001
Systolic blood pressure (mm Hg)	117.3 ± 19.9	118.2 ± 19.3	.77
Diastolic blood pressure (mm Hg)	55.8 ± 9.9	56.1 ± 8.8	.79
Rate-pressure product (mm Hg·minute^−1^)	8,159.5 ± 1,943.0	9,487.0 ± 2,025.4	.004
EDV (mL)	164.5 ± 36.5	162.6 ± 43.9	.59
ESV (mL)	61.8 ± 12.6	62.9 ± 16.0	.64
EF (%)	39.4 ± 10.7	40.6 ± 0.29	.29
Global myocardial perfusion (mL·minute^−1^/100 g)	180.2 ± 59.9	193.6 ± 60.8	.002
Minimal coronary resistance (mmHg·(mL·minute^−1^/100 g)^−1^)	0.49 ± 0.19	0.45 ± 0.16	.038
Global perfusion related to RPP (mL·minute^−1^/100 g)	229.6 ± 96.7	206.0 ± 73.1	.032

The left-ventricular function parameters showed no change (Table 2, middle third).

### Adenosine Side Effects and ECG

The symptom scores during adenosine infusion did not differ significantly, they were 3.7 ± 1.9 with and 3.4 ± 1.6 (*P* = .43) without beta blocker.

ECG changes with ST depressions >0.1 mV occurred in one patient with beta blocker. This patient exhibited more pronounced ST alterations during the 2nd scan. Another patient without ST changes with beta blocker demonstrated ST depressions under adenosine after beta blocker discontinuation.

### Quantitative Analysis

**Figure 1:**
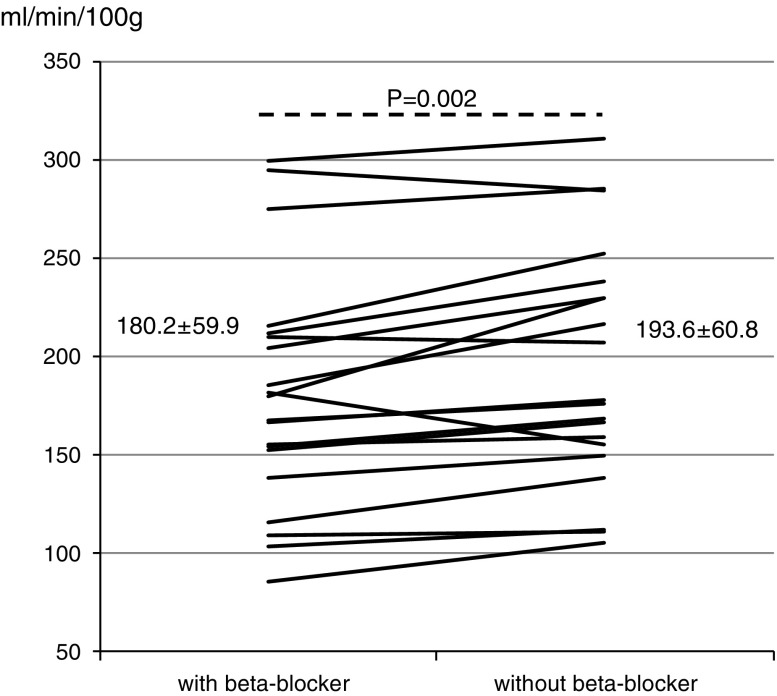
Myocardial perfusion under adenosine with and without beta blocker

**Figure 2:**
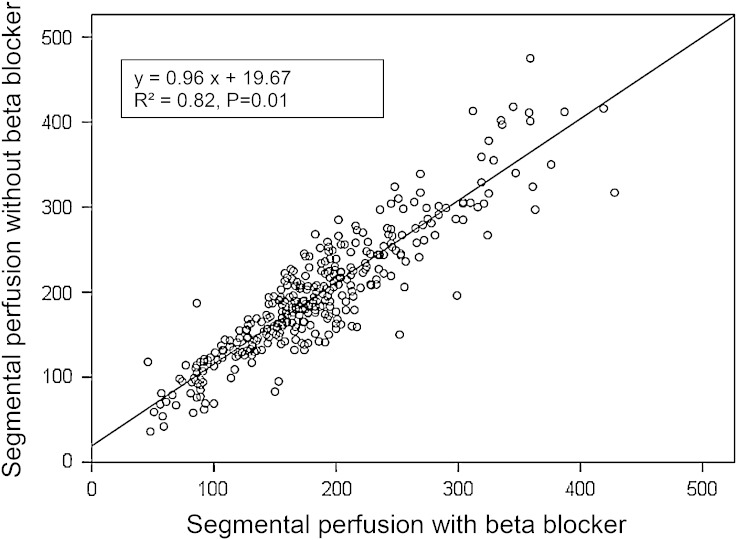
Segmental perfusion with and without beta blocker

The data are listed in Table 2, lower third. Global MBF showed a significant increase by 8% ± 10% (*P* = .002) after beta blocker withdrawal. The individual data are depicted in Figure 1. All but three patients had a lower global MBF without beta blocker than with. The segmental MBF values (Figure 2) demonstrated a strong correlation over the entire range of perfusion values. The average effect was a slight perfusion shift of about 10-15 mL·minute^−1^/100 g in the range of 100-300 mL·minute^−1^/100 g. The mCR under adenosine declined by 5% ± 11% (*P* = .038) and the normalized RPP by 11% ± 21% (*P* = .032) after beta blocker discontinuation.

### PET Study Interpretation

**Figure 3:**
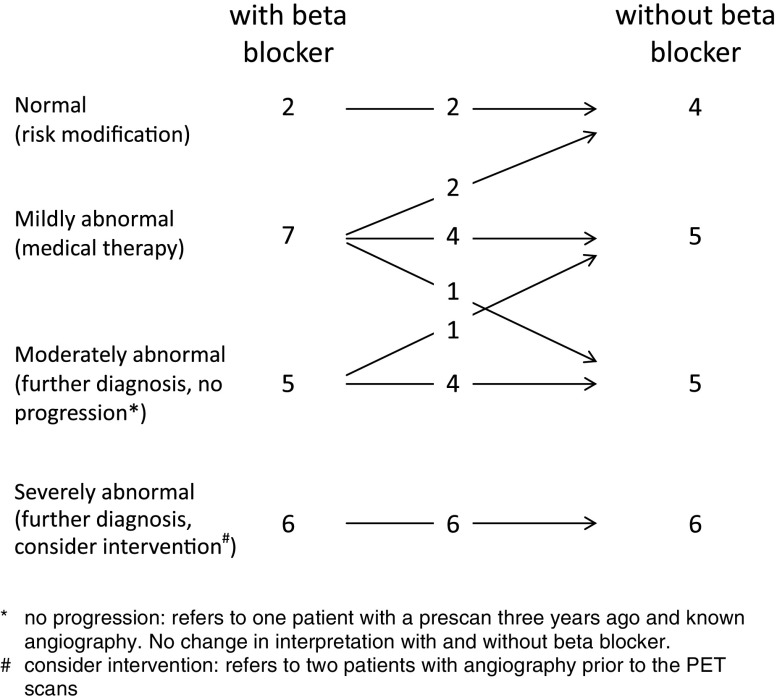
Interpretation of the PET studies and management recommendation

The interpretation of the PET studies with the clinical management recommendation is depicted in Figure 3. Independent of beta blocker intake, all patients with normal and severe MBF abnormalities experienced no change in study interpretation. In four cases, the study interpretation differed by one category. This was in three cases a downstaging, due to the higher MBF after beta-blocker withdrawal and in only one case an upstaging. In two of the four cases an essentially different MPI interpretation, with a change from medical therapy recommendation to angiography or vice versa, was observed. One of these two patients had mild and the other moderate MBF abnormalities.

## Discussion

### Effect of Beta Blocker Withdrawal on Myocardial Perfusion

Beta blockers are one of the most frequent medications in the treatment and management of patients with hypertension and CAD.

The entire mode of action is multiple and still incompletely understood.^23^ Most important in the context of this paper are the negative chronotropic and inotropic effects which decrease cardiac workload and oxygen consumption during exercise stress, and correspondingly MBF. As a consequence, MBF differences required to detect flow-limiting stenosis can be diminished, and thus the diagnostic accuracy of MPI.^24^,^25^ Guidelines therefore recommend a discontinuation of beta blockers for several half-lives before MPI.^13^,^14^

Pathophysiologically, the impact of beta blockers on exercise or dobutamine stress testing is evident.^24^ Their effect on vasodilator stress testing should be minimal since vasodilation occurs uncoupled from oxygen demand. The results of quantitative PET studies addressing this issue, however, are inconsistent:

(1) In 10 healthy volunteers, perfusion under dipyridamole without metoprolol was significantly lower than with (186 ± 27 vs 234 ± 45 mg·minute^−1^/100 g).^26^ (2) In 36 CAD patients with adenosine PET, no effect of metoprolol and carvedilol on global MBF was found, but a significant shift of hyperemic MBF in stenosis-dependent segments.^27^ (3) An increase in hyperemic MBF of about 20% was demonstrated in 14 male CAD patients when metoprolol had been withheld.^11^

In the present study, MBF in CAD patients increased by about 8% over the entire range of measured MBF values after beta blocker discontinuation. As systolic and diastolic blood pressures were not different with and without beta blockade, the increase in heart rate remains as one factor of the MBF shift. In this way, heart rate also indirectly contributes to the decrease in mCR. Of note, heart rate is not the only factor because RPP normalized MBF did not remain constant, but decreased under adenosine. Under resting conditions, the relationship between RPP and MBF was not affected by beta blockade, as reported in former studies.^11^,^26^ Therefore, it is to conclude that further interfering factors are involved in the regulation of MBF under adenosine and beta blockade.

The potential mechanisms are multiple and have been discussed in previous papers in detail: (1) longer diastolic perfusion of the subendothelial layer due to lower heart rate,^11^,^26^ (2) suppression of sympathetic regulation of coronary arteries,^28^ and (3) increase in collateral vessel resistance with fewer poststenotic steal effects.^29^

Changes of the ventricular function parameters EDV and EF were not observed in the present study and can be excluded as causative factors.

However, it needs to be considered that the hyperemic MBF response after beta blocker withdrawal was not uniform in all patients. Three patients (15%) did not exhibit an increase, but a decrease in MBF. This observation supports the aforementioned complex and, according to previous studies, non-uniform interaction between beta blockers and vasodilator MBF.^11^,^26^,^27^

### PET Study Interpretation

Against this background, the question arises of whether, and to what extent, diagnostic accuracy and MPI management recommendation are affected by beta blockers or their withdrawal.

The first issue has been addressed in several studies. Some of them revealed a reduced sensitivity for the detection of flow-limiting CAD.^9^,^10^,^12^ Sharir et al^9^ studied 21 patients with and without their individual antianginal medication (21 patients with calcium antagonists, 19 with nitrates, and 8 with beta blockers) and found a sensitivity of 92% without and 62% with medication. Taillefer et al considered placebo vs metoprolol with dipyridamole MPI. Sensitivity decreased from 85.7% with placebo to 71.4% with metoprolol. Of note, 17 patients out of 21 had similar results with placebo and metoprolol, but only four differed.^10^ Reyes et al^12^ demonstrated in 45 patients a small but significant reduction in the extent and severity of perfusion abnormalities under adenosine, and a decrease in sensitivity from 76% to 58% under beta blockade. Yoon et al^15^ retrospectively studied 555 patients with MPI and angiography and found similar sensitivities between those with and without beta blockers and similar summed stress scores. Likewise, Lakkireddy et al^16^ (158 patients, 48 with beta blocker withdrawal) revealed no impact of beta blockade on extent, severity, or reversibility of perfusion defects.

In the present study, we considered the effect of beta blockers and their withdrawal on the MPI result and its interpretation.

The interpretation matrix of the PET studies (Figure 3) shows that MPI results and management recommendation were equivalent in the majority (80%), irrespective of beta blocker intake and associated MBF changes. In 10% the higher MBF after beta blocker withdrawal led to mild changes in terms of a downstaging, but without an essential impact on the interpretation. To summarize, in a total of 90% of our study patients the MPI results did not basically differ independent of beta blocker intake, particularly in high-risk and normal MPI patterns. Only 10% presented an essential change in study interpretation. This subgroup needs further characterization. The patient number of this study is, however, too small for a deeper analysis.

## New Knowledge Gained

- In CAD patients, the withdrawal of a beta blocker is associated with a significantly higher MBF under adenosine, 8% on average.
- This MBF shift is found over the entire range of measured MBF values.
- Heart rate is one factor of this increase, but further interfering factors are involved.
- MPI interpretation and individual management recommendation are, in the majority of cases, particularly in normal and high risk MPI patterns, independent of beta blocker intake prior to the stress test.
- In a few patients, the higher MBF without beta blocker results in a downstaging in MPI interpretation and management recommendation.
- Notably, there is a small CAD patient subgroup which differs from these patterns and which needs further investigation in a larger study cohort.

## Conclusion

The question of whether beta blockers need to be stopped before vasodilator stress testing cannot be answered for a diverse CAD population with a universal “no” or “yes.” As adenosine MBF without beta blockers is higher than with, the clinical pathway recommending the stop of beta blockers prior to stress testing in order to ensure the highest MBF remains advisable. However, if a temporary beta blocker withdrawal is unfeasible due to complications, contraindications, a tight clinical schedule, or because a patient simply forgot to withhold the medication, it is appropriate to perform adenosine stress testing in such cases.

### Study Limitations

In this study, patients with known CAD, comorbidities, and cardiac co-medication were considered. The results may not be directly transferable to low or moderate risk groups scheduled for exclusion of CAD with MPI.

The cardiac co-medication may have interfered with perfusion to a different extent between both adenosine studies. A potential bias is not assessable. By leaving the co-medication unchanged between the studies, an attempt was made to minimize any co-medication effects.

The patients took a total of four different beta blockers, three of them cardioselective. An optimal study condition would have been with one single beta blocker in all patients. However, 95% of the patients had cardioselective beta blockers with comparable working profiles. Thus, a confounding effect by the different beta blockers is thought to be low.

The overall sample size of this studied population is very small. Thus, it is very difficult to draw hard conclusions from the study. The results in Figures 1 and 3 show that the withdrawal of beta blockers leads to divergent and variable changes in MBF and thus limits its application in clinical decision making. This also raises the question of whether the observations seen are more random and not systematic in nature. For all these reasons, a larger study is clearly needed.
